# Supplementary material for: Morin-VitaminE-β-CyclodextrinInclusionComplexLoadedChitosanNanoparticles (M-Vit.E-CD-CSNPs) Ameliorate Arsenic-Induced Hepatotoxicityina Murine Model
Source: Molecules. 2022 Sep 8;27(18):5819. doi: 10.3390/molecules27185819 (PMC9504860; doi:10.3390/molecules27185819)
Supplement: Supplementary file 1 [file molecules-27-05819-s001.zip › molecules-1774356-supplementary.pdf]

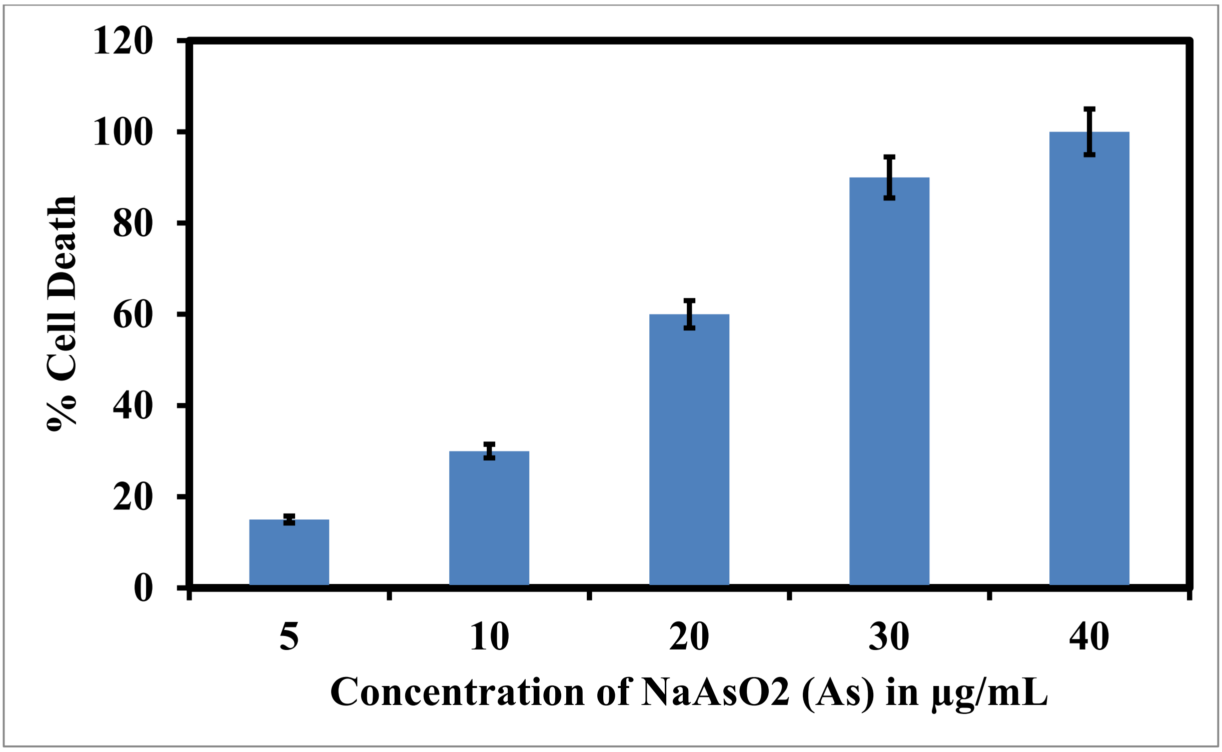

Figure S1: HepG2 (human hepatocellular carcinoma) cells ($1x10^6$ cells/mL) were treated with different concentrations of sodium arsenite ranging from 5 to 40µg/mL for 24 h. % cell death values were calculated from an MTT assay. Results presented here are one of the three representative experiments ±S.D.

Figure S2:

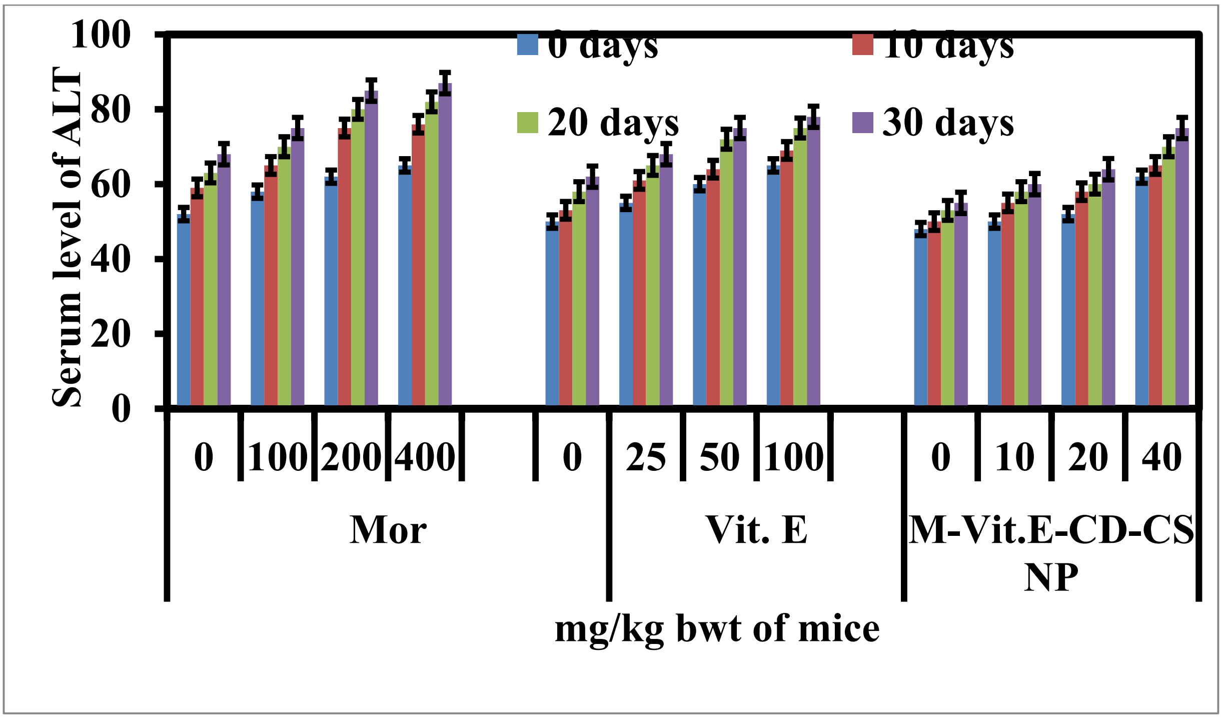

Figure S3:

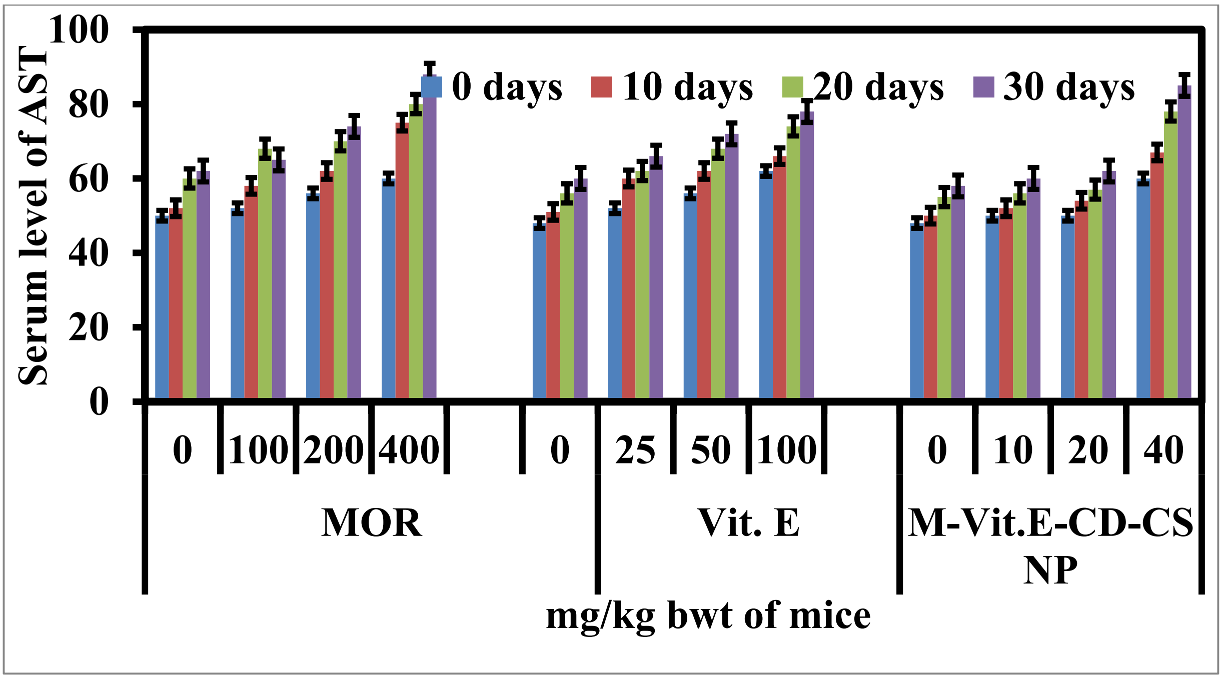

Figure S2 and S3: Effect of Morin (MOR), vitamin E and M-Vit.E-CD-CS NPs on liver function markers (ALT and AST). Different dosage of Morin (MOR), vitamin E and M-Vit.E-CD-CS NPs

were orally given to the normal BALB/c mice and the serum level of ALT and AST were measured. Data is one of the three representative experiments ± SD.

Table S1: Effect of oral administration of MOR and MCNPs on haematological Parameters

Parameters	Control Group	Arsenic (40 mg/L) treated mice	Arsenic + MOR (200 mg/kg) treated mice	Arsenic + Vitamin E (50 mg/kg) treated mice	Arsenic + M-Vit.E-CD-CS NPs (20 mg/kg)
**Body weight gain (gm)**	**0.52 ± 0.06**	**0.29 ± 0.03**	**0.45 ± 0.04**	**0.39 ± 0.02**	**0.49 ± 0.03**
**RBC : No. of cells (10^6/µL)**	**8.1 ± 0.53**	**5.2 ± 0.35**	**6.8 ± 0.27**	**6.1 ± 0.33**	**7.5 ± 0.41**
**WBC : No. of cells (10^3/µL)**	**12.6 ± 0.13**	**16.2 ± 0.31**	**15.1 ± 0.24**	**15.8 ± 0.41**	**13.1 ± 0.22**
**Hb (gm/dl)**	**13.6 ± 0.38**	**11.7 ± 0.23**	**12.5 ± 0.47**	**12.1 ± 0.17**	**12.9 ± 0.38**
**PLT (10^3/µL)**	**556 ± 31.2**	**412 ± 28.2**	**512 ± 25.8**	**516 ± 36.2**	**545 ± 19.7**
**LDH (U/L)**	**401 ± 24.3**	**742 ± 31.3**	**495 ± 14.5**	**481 ± 16.6**	**412 ± 11.3**
**Uric acid (mg/dL)**	**2.61 ± 0.72**	**4.73 ± 0.51**	**3.09 ± 0.34**	**3.21 ± 0.51**	**2.76 ± 0.42**
**Creatinine (mg/dL)**	**0.51 ± 0.04**	**2.8 ± 0.13**	**1.7 ± 0.09**	**1.5 ± 0.06**	**0.61 ± 0.03**
**Cholesterol (mg/dL)**	**145 ± 7.6**	**281 ± 31.2**	**162 ± 11.2**	**173 ± 16.3**	**151 ± 4.5**
**TG (mg/dL)**	**85.1 ± 8.2**	**163 ± 12.1**	**92.4 ± 9.1**	**104.4 ± 6.2**	**89.3 ± 3.8**
**HDL (mg/dL)**	**61.8 ± 2.9**	**35.6 ± 4.1**	**45.1 ± 2.3**	**48.4 ± 3.1**	**55.3 ± 3.5**
**LDL (mg/dL)**	**76.2 ± 5.7**	**165.6 ± 10.8**	**92.5 ± 6.1**	**95.6 ± 5.3**	**81.1 ± 4.6**
**Phospholipid (mg/dL)**	**45.5 ± 7.5**	**22.1 ± 4.3**	**38.1 ± 2.9**	**35.3 ± 3.1**	**42.5 ± 3.1**

Values are expressed as mean ± SEM (n=3). P>0.05 when compared to normal group.

Table S2: Arsenic deposition in different organ

	Arsenic concentration in µg/g of tissue in 30 days					
	**Liver**	**Kidney**	**Cerebellum**	**Lung**	**Heart**	**Skin**
**Arsenic, 40 mg/L**	**145.3 ± 5.1**	**29.8 ± 1.8**	**12.5± 4.4**	**14.6 ± 1.3**	**12.8 ± 5.6**	**4.8 ± 2.6**
**Arsenic + MOR (200 mg/kg)**	**110.4 ± 2.5**	**18.1 ± 1.4**	**8.2 ± 2.1**	**9.9 ± 1.9**	**8.5 ± 2.2**	**1.9 ± 0.6**
**Arsenic + vitamin E (50 mg/kg)**	**95.2 ± 2.4**	**15.1 ± 1.6**	**6.6 ± 1.3**	**7.1 ± 1.1**	**6.1 ± 0.8**	**0.7 ± 0.16**
**Arsenic + M-Vit.E-CD-CS NPs (20 mg/kg)**	**41.6 ± 3.3**	**3.1 ± 1.2**	**0.52 ± 0.07**	**1.2 ± 0.03**	**1.5 ± 0.21**	**0**

Values are expressed as mean ± SEM (n=3). P>0.05 when compared to normal group.
